# TSH Combined with TSHR Aggravates Diabetic Peripheral Neuropathy by Promoting Oxidative Stress and Apoptosis in Schwann Cells

**DOI:** 10.1155/2021/2482453

**Published:** 2021-11-11

**Authors:** Jingwen Fan, Qi Pan, Qun Gao, Wenqing Li, Fei Xiao, Lixin Guo

**Affiliations:** ^1^Peking University Fifth School of Clinical Medicine, Beijing, China; ^2^Department of Endocrinology, Beijing Hospital, National Center of Gerontology, Beijing, China; ^3^Department of Neurosurgery, Beijing Hospital, National Center of Gerontology, Beijing, China; ^4^The Key Laboratory of Geriatrics, Beijing Institution of Geriatrics, Beijing Hospital, National Center of Gerontology, National Health Commission, Institute of Geriatric Medicine, Chinese Academy of Medical Sciences, Beijing, China

## Abstract

Subclinical hypothyroidism (SCH) is associated with diabetic peripheral neuropathy (DPN); however, the mechanism underlying this association remains unknown. This study is aimed at examining neurofunctional and histopathological alterations in a type 2 diabetes (T2DM) mouse model of SCH and investigating the impact of thyroid-stimulating hormone (TSH) in an in vitro DPN cell model established using RSC96 cells under high glucose (HG) and palmitic acid (PA) stimulation. Our results indicated that T2DM, in combination with SCH, aggravated abnormal glucose and lipid metabolism in T2DM and dramatically destroyed the peripheral nervous system by increasing paw withdrawal latency, decreasing motor nerve conduction velocity, and exacerbating ultrastructural deterioration of the damaged sciatic nerve caused by diabetes. Furthermore, the results of our in vitro experiments showed that TSH intensified HG/PA-induced RSC96 cell damage by inducing oxidative stress, mitochondrial dysfunction, and apoptosis. More importantly, TSHR knockout or inhibition of PA-induced TSHR palmitoylation could alleviate the apoptosis induced by TSH. Overall, in this study, the novel mechanisms by which TSH, as an independent risk factor for DPN progression, aggravating Schwann cell apoptosis and demyelination, are elucidated. These findings indicate that TSHR could be a potential target for both the prevention and treatment of DPN and, possibly, other microvascular diseases, and have implication in the clinical management of patients with DPN.

## 1. Introduction

Diabetic peripheral neuropathy (DPN), which affects approximately 50% of patients with diabetes, is the most frequently encountered complication of long-term diabetes [1, 2]. It may lead to significant morbidity, sensory dysfunction, pain, and a high risk of falling and is, thus, associated with a decline in quality of life. Further, DPN pathogenesis, which is characterized by the destruction of neurons and Schwann cells, as well as microvessels inside nervous fibers, is complicated and remains unclear, despite extensive research on this condition [3]. Hyperglycemia [4], dyslipidemia, rising serum uric acid levels [5], smoking [6], and vitamin D deficiency [7] have also been described as factors that contribute to the development of DPN.

The majority of studies on DPN have focused on the impact of this disease on neurons, while studies with a focus on Schwann cells, which are the predominant cells in the peripheral nervous system (PNS), are limited [8]. Reportedly, Schwann cells play an important role in the growth and regeneration of the PNS. Specifically, they are essential for maintaining neuronal structure and function and for nourishing axons and promoting survival and repair after injury. Given that the first pathological process of DPN is segmental axonal demyelination and myelin regeneration followed by axonal degeneration, it is plausible to think that Schwann cell damage may underlie nerve fiber injury and possibly represent the first step in DPN pathogenesis. Furthermore, the mechanism of Schwann cell involvement in DPN pathogenesis is considered to be sustained hyperglycemia, which leads to increased fluxes of polyol pathways in Schwann cells; a decrease in the production of neurotrophic factors, such as myelin, ciliary neurotrophic factor (CNTF), nerve growth factor (NGF), and neurotrophin-3 (NT-3); mitochondrial dysfunction; oxidative stress; altered lipid metabolism; the release of inflammatory factors that bring about neuroinflammation; axonal demyelination; and slow nerve conductive velocity (NCV), which in turn promotes DPN progression [8].

Thyroid dysfunction and type 2 diabetes mellitus (T2DM) are relatively common endocrine diseases that often appear together. Specifically, subclinical hypothyroidism (SCH), which is characterized by elevated plasma thyroid-stimulating hormone (TSH) levels and normal free thyroxine (FT4) and triiodothyronine (FT3) levels, is the most frequently encountered thyroid disease [9, 10], which patients with T2DM are more likely to develop compared with the general population. A meta-analysis that included 17 studies showed that the prevalence of SCH in patients with T2DM varied in the range 4.69~18.86% [11]. Furthermore, owing to the absence of typical SCH symptoms, the condition is often easily ignored in clinical work. Thus, its potential hazards have gradually attracted the attention of researchers.

Patients with T2DM and SCH are more likely to suffer from DPN. A systematic review and meta-analysis showed that SCH is an independent risk factor that can be used to predict the development of DPN, with a hazard ratio (95% CI) of 1.87 (95% CI: 1.06, 3.28) [11]. Additionally, other studies have also shown that the TSH level, which is an independent risk factor for neuropathy in T2DM, is significantly associated with DPN [12, 13]. However, there is currently little fundamental research on DPN with SCH, and the mechanism by which SCH exacerbates DPN is still unknown. Therefore, given that TSH is the only thyroid hormone at the abnormal range in SCH, we hypothesized that it affects DPN progression by enhancing Schwann cell oxidative stress and apoptosis.

To verify this hypothesis, first, we established a mouse model of T2DM with SCH to investigate the effect of SCH on DPN in vivo. Second, we treated the rat Schwann cell line RSC96 with high glucose (HG) and a high concentration of palmitic acid (PA) in vitro to mimic glucolipotoxicity in vivo and explore the effects of TSH on oxidative stress and apoptosis, as previously reported [14]. Additionally, given that the function of TSH is mediated by the TSH receptor (TSHR), we also investigated the effect of the presence of TSHR in RSC96 cells on DPN progression. To our best knowledge, this study is the first to demonstrate that Schwann cells express functional TSHR and that diabetes with SCH exacerbates DPN, possibly through a mechanism wherein TSH increases oxidative stress and proapoptotic effects in Schwann cells.

## 2. Materials and Methods

### 2.1. Animals

This study was approved by the Biomedical Ethics Committee of Peking University (approval number No. LA2021426) and was conducted in compliance with the guidelines set forth by the Declaration of Helsinki. C57BL/6 mice were purchased from Huafukang (Beijing, China). All the animals were kept in a temperature-controlled environment (22–23°C) with a 12 h/12 h light/dark cycle. To establish the T2DM mouse model, 6-week-old mice were fed a high-glucose and high-fat diet (HFD), containing 10% lard oil, 20% sucrose, 0.5% sodium cholate, and 2.5% cholesterol, for 4 weeks and then intraperitoneally injected with streptozotocin (STZ, 50 mg/kg body weight) dissolved in 0.1 M citrate buffer at a pH of 4.4 or administered a comparable volume of vehicle for five consecutive days. After this treatment, the tail blood glucose levels in mice were measured using a glucometer (Sinocare, Hunan, China), and mice with fasting blood glucose (FBG) > 16.7 mmol/L were considered diabetic. At 11 weeks, both normal and diabetic mice were divided randomly into two groups. The mice in one group were administered methimazole (0.08 mg/kg BW/d) via drinking water for 14 weeks to establish a mouse model of SCH as previously described [15], while those in the other group were treated with normal water. All the mice were sacrificed, at 25–26 weeks of age, under isoflurane anesthesia. To confirm successful establishment of the SCH model, blood samples were collected to detect FT3, FT4, and TSH levels.

The oral glucose tolerance test (OGTT) was performed, and the area under the curve (AUC) was determined to quantify results from the OGTT test. Furthermore, glycated hemoglobin (HbA1c) levels were quantified enzymatically using an ELISA kit (Jiancheng Bioengineering Institute, Nanjing, China).

### 2.2. Hot Plate Test

During the terminal stage of the disease, we performed a hot plate test on the mice to evaluate their paw withdrawal latency (PWL) using a GL-150B system (Haimen KylinBell Lab Instruments Co., Ltd., Jiangsu, China). The temperature for the test was set at 56°C.

### 2.3. Evaluation of Motor Nerve Conduction Velocity

Motor nerve conduction velocity (MNCV) was measured using a Dantec Keypoint Focus EMG Monitor (Skovlunde, Denmark). Briefly, the mice were anesthetized, and their left sciatic nerves were exposed. Thereafter, the exposed sciatic nerve trunks were stimulated via bipolar recording electrodes (duration, 0.1 ms; intensity, 3.5 mA), and the action potential at the gastrocnemius muscle, abdomen, or toe was recorded. Thereafter, the MNCV was calculated according to the following equation.

(1) $$\begin{array}{c} \text{MNCV }\left(\text{m}/\text{s}\right)=\frac{\text{Distance between the stimulating and recording electrodes}}{\text{Latency}}. \end{array}$$

### 2.4. Morphological Observation of Sciatic Nerve

Histopathological examination and transmission electron microscopy (TEM) observations were performed to investigate morphological alterations in the sciatic nerves. Specifically, the sciatic nerves were stained with hematoxylin eosin (H&E) and photographed using an inverted microscope (IX71; Olympus, Tokyo, Japan). Furthermore, for TEM, the sciatic nerve was excised and fixed with 2.5% glutaraldehyde in 0.1 M phosphate buffer (pH 7.4) at 4°C overnight, followed by postfixation in 0.1 M osmium tetroxide for 2 h. Thereafter, the specimens were dehydrated and embedded in epoxy resin. Ultrathin slices were cut and stained with uranyl acetate and lead citrate. The sections were then photographed and analyzed using a TEM-1400 Plus transmission electron microscope (JEOL Ltd., Tokyo, Japan).

### 2.5. Mouse Thyroid Function and Blood Lipid Level Determination

Plasma FT3, FT4, and TSH levels were detected using Elabscience Biotechnology assay kits (Wuhan, China) according to the manufacturer's instructions. Furthermore, the levels of different lipids, including total cholesterol (TC), triglyceride (TG), low-density lipoprotein cholesterol (LDL-C), and high-density lipoprotein cholesterol (HDL-C), in the blood samples of mice were determined according to the manufacturer's instructions (Nanjing Jiancheng Bioengineering Institute, Nanjing, China). Additionally, serum insulin, adiponectin, and leptin levels were analyzed using an ELISA kit (R&D Systems, Minneapolis, MN, USA), and the homeostatic model assessment for insulin resistance (HOMA-IR) was performed according to the following equation.

(2) $$\begin{array}{c} \text{HOMA}‐\text{IR}=\frac{\text{FBG}\times \text{insulin}}{22.5}. \end{array}$$

### 2.6. Immunohistochemistry and Immunofluorescence

Sciatic nerves were fixed with 4% paraformaldehyde and embedded in paraffin. To perform immunohistochemistry (IHC) analysis, the samples were dewaxed in xylene, dehydrated in alcohol, and microwaved at 95°C for 3 min in citric saline for antigen retrieval. Thereafter, they were treated with 3% H_2_O_2_ for 25 min, and after blocking with 5% bovine serum albumin (BSA), sections were incubated overnight with an appropriate primary antibody at 4°C followed by incubation with the secondary antibody for 1 h. Sections were then photographed using an optical microscope (Eclipse E200, Nikon, Japan). Additionally, for immunofluorescence (IF) analysis, slices were incubated with Cy3- or FITC-conjugated secondary antibodies. The stained specimens were observed and captured using a confocal microscope (Eclipse Ti, Nikon, Tokyo, Japan). The antibodies for IHC and IF, i.e., rabbit polyclonal anti-TSHR (1 : 50, 14450-1-AP), rabbit polyclonal anti-Caspase 3 (1 : 50, 19677-1-AP), and rabbit polyclonal anti-S100 (1 : 50, 15146-1-AP), were purchased from Proteintech (Chicago, USA).

### 2.7. Cell Culture and Treatment

The RSC96 cell line was established via spontaneous transformation of long-cultured primary cultures of rat Schwann cells. RSC96 cells (ATCC) and HEK293T cells (Type Culture Collection of the Chinese Academy of Medical Sciences) were cultured in DMEM containing 10% fetal bovine serum (FBS, Gibco, Waltham, MA, USA), while a normal human thyroid follicular epithelial cell line (Nthy-ori 3-1 cells, Stem Cell Bank/Stem Cell Core Facility) was cultured in RPMI-1640 medium (Hyclone, South Logan, UT, USA) supplemented with 10% FBS. Furthermore, the medium used for producing lentivirus using HEK293T cells was DMEM supplemented with 30% FBS. All the cells were cultured at 37°C in 5% CO_2_. After a 12 h incubation in a serum-free medium, the cells were treated with or without HG and PA and with or without TSH (ProSpec-Tany TechnoGene Ltd., Rehovot, Israel). PA was solubilized in 0.1 M NaOH and mixed with 20% BSA. Mannitol was used to match the hyperosmolality of the cells cultured under hyperglycemic conditions.

### 2.8. TSHR Knockout RSC96 Cells

To produce single-guide RNA (sgRNA), the CHOPCHOP design tool (http://chopchop.cbu.uib.no/) was used to select a target DNA sequence that precedes a 5′-NGG sequence in the genomic *TSHR* locus (NM_012888.2). The target sequence was TGGAGGTCCCTTGGAAAAT. Primer and oligo synthesis and PCR product sequencing were performed by Sangon Biotech (Shanghai, China).

After digesting the lentiCRISPR v2 plasmid (3 *μ*g) with 1 *μ*L BsmB1-v2 enzyme at 55°C for 1 h, the correct-sized fragment was verified by running the samples on a 0.8% agarose gel. The ligation reaction for sgRNA was then set up. This was followed by incubation at 16°C for 1 h and cloning into Trans5*α* chemically competent cells (TransGen Biotech, Beijing, China). Additionally, to confirm the insertion of sgRNA into the lentiCRISPR v2 vector, plasmid DNA was isolated from bacterial cultures using the TIANprep Mini Plasmid Kit (Tiangen Biotech, Beijing, China).

The sgRNA-lentiCRISPR vector (2.5 *μ*g), the packaging plasmid psPAX2, and the envelope plasmid pMD2.G (Hanbio Biotechnology Co. Ltd., Shanghai, China) were mixed with 4 *μ*L of Lipofectamine 3000 (Thermo Fisher Scientific, Waltham, MA, USA) in 125 *μ*L Opti-MEM (Thermo Fisher Scientific). The mixture was then added to HEK293T cells, and the viral supernatant was collected at 72 h posttransfection and centrifuged to remove cell debris. The filtered supernatant was then titrated to infect the RSC96 cells. Finally, the infected cells were cultured in media containing 4 *μ*g/mL puromycin (Bioruler, Danbury, CT, USA), for 4 weeks. Thereafter, the samples were subjected to sequencing or western blot analysis to confirm TSHR expression.

### 2.9. RNA Extraction and PCR

Total RNA was extracted from RSC96 cells using a TRIzol reagent (Invitrogen, Waltham, MA, USA). The concentration and purity of total RNA were analyzed using a NanoDrop 2000 instrument (Thermo Fisher Scientific, Waltham, MA, USA). cDNA was generated using the M-MLV Reverse Transcriptase Kit (Invitrogen). The primer sequences used are listed in Table 1. The TSHR DNA was amplified via PCR, and the products were detected on an agarose gel.

### 2.10. cAMP Assay

RSC96 cells were incubated in a serum-free medium containing 0.5 mM 3-isobutyl-1-methylxanthine (IBMX, Sigma) alone, as a cAMP phosphodiesterase inhibitor, or in combination with TSH for 1 h at 37°C. Forskolin (MedChemExpress, New Jersey, USA), an adenylyl cyclase (AC) activator, was used as the positive control. After incubation, the cell lysates were collected to detect intracellular cAMP levels using an ELISA kit (KGE002B, R&D Systems) according to the manufacturer's protocol.

### 2.11. Cell Counting Kit-8 (CCK-8) Assay

RSC96 cells were cultured in 96-well plates, at a density of 5000 cells per well, and treated with different concentrations of glucose or PA for 24, 48, 72, or 96 h. Thereafter, 10 *μ*L of the CCK-8 solution (Beyotime Biotechnology, Shanghai, China) was added to each well, followed by incubation at 37°C for 1 h. The optical density (OD) at 450 nm was measured using a Synergy H1 microplate reader (BioTek, Vermont, VT, USA). The experiments were performed in triplicates.

### 2.12. Cell Apoptosis Assay

Given that apoptotic cells exhibit apoptotic bodies and enhanced chromatin condensation, Hoechst 33342 dye was used to assess chromatin condensation in the RSC96 cells. Specifically, RSC96 cells were collected and stained with Annexin V and propidium iodide (PI) using an apoptosis kit (KeyGen Biotech, Nanjing, China). Thereafter, 1 × 10^5^ cells were suspended in 500 *μ*L of binding buffer containing 5 *μ*L Annexin V-FITC and 10 *μ*L PI. This was followed by incubation in the dark for 15 min, after which the cells were subjected to flow cytometry using a FACSCanto system (BD Biosciences, San Jose, CA, USA) and analyzed using the FlowJo software (Tree Star Inc., Ashland, OR, USA). At least three independent experiments were performed. Furthermore, TUNEL staining was performed on the RSC96 cells using the KeyGen kit (KeyGen Biotech), and apoptotic cells were observed using a confocal microscope and quantified by counting the positively stained cells from five random fields in three independent experiments.

### 2.13. Cellular and Mitochondrial ROS Production

The levels of intracellular reactive oxygen species (ROS) were measured using a DCFH-DA fluorescent probe (Beyotime Biotechnology, Shanghai, China). The cells were treated with 10 *μ*M DCFH-DA at 37°C for 30 min, followed by fluorescence intensity examination using a confocal fluorescence microscope. Further, to determine the degree of mitochondrial oxidative stress, RSC96 cells were incubated with 50 *μ*M of MitoSOX™ Red (Invitrogen, Waltham, MA, USA) at 37°C for 15 min. Pictures were captured immediately after washing with PBS buffer.

### 2.14. Mitochondrial Membrane Potential Determination

Mitochondrial membrane potential (MMP) was evaluated using the JC-1 assay kit (Beyotime Biotechnology, Shanghai, China). Briefly, RSC96 cells were incubated with JC-1 in the absence of light at 37°C for 20 min. Given that the fluorescence intensity ratio (red/green) declines in the presence of mitochondrial depolarization, after washing twice, the cells were observed under a confocal microscope.

### 2.15. Western Blot Analysis

After washing with ice-cold PBS, cells in a 6-well-plate were lysed in lysis buffer (1 mM PMSF and phosphatase inhibitors) (Beyotime Biotechnology, Shanghai, China). After centrifugation at 12000 g for 15 min, the supernatant was collected, mixed with 5x protein loading buffer, and boiled for 15 min. The proteins were separated via sodium dodecyl sulfate polyacrylamide gel electrophoresis (SDS-PAGE), transferred onto polyvinylidene difluoride (PVDF) membranes (Merck Millipore, Billerica, MA, USA), and subjected to western blot analysis (WB) using the corresponding antibodies. Finally, the bands were visualized with a ChampChemi gel imager (Sage creation, Beijing, China) using Western Chemiluminescent HRP Substrate (Millipore). Band densities of target proteins were normalized to those of *β*-actin or GAPDH. Rabbit polyclonal anti-GAPDH (1 : 2500, ab9485) and mouse monoclonal anti-*β*-actin (1 : 10000, ab49900) were purchased from Abcam (Cambridge, UK). The following antibodies were purchased from Proteintech: rabbit polyclonal anti-Caspase 3 (1 : 500, 19677-1-AP), rabbit polyclonal anti-Caspase 9 (1 : 500, 10380-1-AP), rabbit polyclonal anti-Bcl2 (1 : 1000, 12789-1-AP), mouse monoclonal anti-Bax (1 : 3000, 60267-1-AP), and rabbit polyclonal anti-TSHR (1 : 500, 14450-1-AP). Three independent experiments were conducted, and signal intensities were determined via densitometry using the ImageJ software (NIH, Bethesda, MD, USA).

### 2.16. ATP Production Measurement

The intracellular ATP concentration was determined using an ATP assay kit (S0026, Beyotime, Shanghai, China). Briefly, RSC96 cells were lysed and centrifuged to acquire protein extracts, which were then added to the reaction buffer according to the manufacturer's instructions. Luminescence values (unit: RLU) were then measured using a Synergy H1 microplate reader (BioTek, Winooski, VT, USA). The ATP content was normalized to the protein content in each sample.

### 2.17. Determination of Malondialdehyde (MDA)

RSC96 cells were seeded in 6-well plates. Thereafter, the cells were lysed and centrifuged, and the supernatant was mixed with MDA solution and heated for 15 min according to the manufacturer's instructions for MDA test kits (Beyotime, Shanghai, China). The absorbance was measured at 532 nm, and the MDA content was normalized to the protein content in each sample.

### 2.18. S-Palmitoylation Assay

We performed the S-palmitoylation assay according to previously described protocols [16] with some minor modifications. In brief, RSC96 cells were lysed in a lysis buffer (10 mM sodium phosphate, 2 mM Na_2_-EDTA, 0.32 M sucrose, 1% Triton X-100, 50 mM *N*-ethylmaleimide, protease, and phosphatase inhibitor) for 30 min. After blocking free sulfhydryl groups with 50 mM N-ethylmaleimide as well as protease and phosphatase inhibitors, the supernatants were immunoprecipitated using a protein A/G resin preloaded with TSHR antibody at 4°C overnight. Thereafter, the protein A/G resin was washed three times and incubated with the elution buffer (1% SDS, 10 mM sodium phosphate, 2 mM Na_2_EDTA, and 0.32 M sucrose) at 50°C for 5 min to release TSHR. The eluted samples were then divided into two equal portions. One portion was incubated with 1 M hydroxylamine and thiol-Sepharose 6B, while the other, which served as the control, was incubated with 1 M Tris·HCl (pH 7.4) and thiol-Sepharose 6B (Sigma-Aldrich, St Louis, MO, USA; Merck KGaA, Darmstadt, Germany) for 2 h. The Sepharose beads were washed three times with the washing buffer (10 mM sodium phosphate, 2 mM Na_2_EDTA, 0.32 M sucrose, 1% Triton X-100, 500 mM NaCl, and 0.2% SDS) followed by WB to determine the presence of TSHR. Thereafter, we submitted the TSHR sequence to predict potential lipid modification of cysteines using GPS-Lipid (http://lipid.biocuckoo.org/index.php).

### 2.19. Statistical Analysis

All data were analyzed using SPSS software v21.0, (IBM Corp., Armonk, NY, USA) and GraphPad Prism 6.0 (GraphPad Software, Inc., La Jolla, CA, USA). The data obtained were presented as mean ± standard deviation (SD) of at least three independent experiments. Two-group differences were analyzed using Student's *t*-test, and intergroup comparisons were performed via a one-way analysis of variance (ANOVA). The Tukey method was used for multiple comparisons via ANOVA. When homogeneity of normal and variance was not satisfactory, the Kruskal-Wallis test was selected; additionally, Dunn's multiple comparison test was performed. Statistical significance was set at *P* < 0.05.

## 3. Results

### 3.1. Abnormal Glucose and Lipid Metabolism in T2DM Mouse Model of SCH

**Figure 1:**
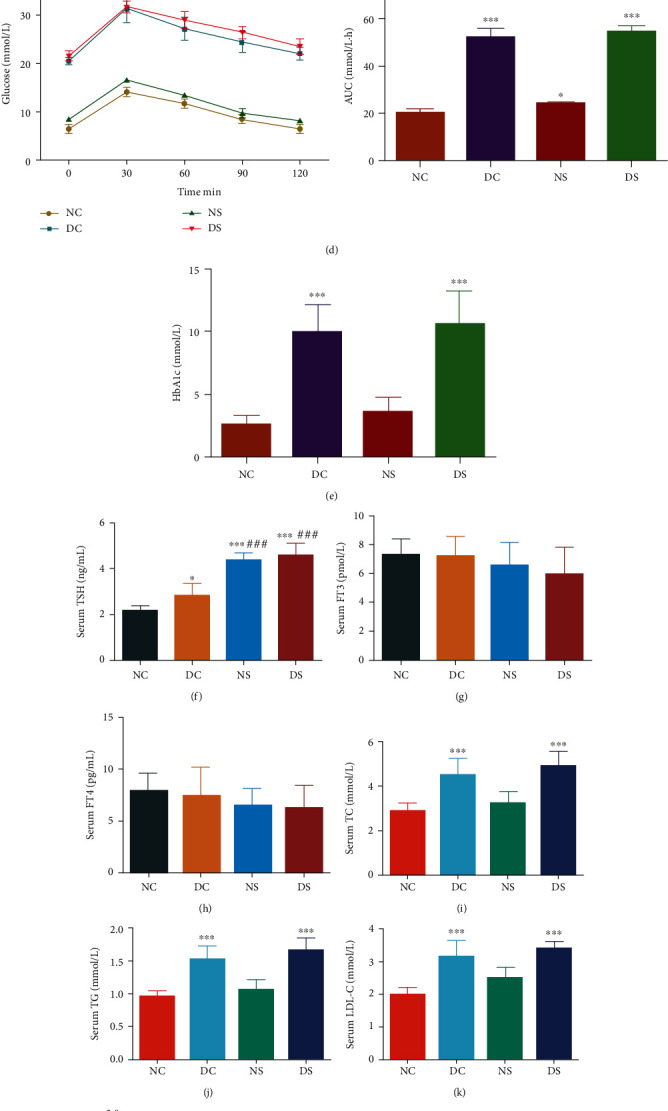
Diabetic mice with SCH showing more severe glycolipid abnormality than diabetic mice without SCH. (a) The proposed mice groups and protocols. NC: normal control (*n* = 7); DC: diabetic control (*n* = 7); NS: normal SCH induction (*n* = 7); DS: diabetic SCH induction (*n* = 7). (b) Body weight of mice. SCH induction increased the body weights of both control and diabetic mice. (c) Serum fasting blood glucose (FBG) levels and (d) curve showing the variation in oral glucose tolerance test (OGTT) results with time. T2DM mice showed an abnormal glucose tolerance curve, with SCH induction enhancing this abnormal glucose metabolism. (e) HbA1c levels in mice. Serum (f) TSH, (g) FT3, and (h) FT4 levels. (i–l) Lipid levels corresponding to the four mice groups. Serum (m) leptin, (n) adiponectin, (o) insulin levels, and (p) HOMA-IR in mice. Blood glucose homeostasis was disturbed in both DC and NS mice. SCH: subclinical hypothyroidism. All data are presented as mean ± SD; a one-way ANOVA was used to analyze (b, d, f, g, h, m–o), and nonparametric Kruskal-Wallis test was used to analyze (c, e, i–l, p) (^∗^*P* < 0.05, ^∗∗^*P* < 0.01, and ^∗∗∗^*P* < 0.001, compared with the NC mice; ^#^*P* < 0.05, ^##^*P* < 0.01, and ^###^*P* < 0.001, compared with DC mice).

To investigate the impact of TSH on diabetic PNS, we established a T2DM mouse model of SCH to replicate the combination of SCH and T2DM, similar to that in humans.  Figure 1(a) depicts the timeline of the experiments involving the T2DM mice with induced SCH. Four mice groups, (i) normal mice drinking normal water (NC), (ii) diabetic mice drinking normal water (DC), (iii) normal mice drinking water containing methimazole (NS), and (iv) diabetic mice drinking methimazole water (DS), were established. Experimental measurements (hot plate test and neurotic electrophysiology) were performed at 24 weeks after the initial classification of the mice into the different groups. Mice in the NC group were fed a normal diet and injected with the vehicle treatment at the age of 11 weeks, while mice in the DC group were fed a high-fat diet for 4 weeks and injected with STZ at the age of 11 weeks. Further, mice in the NS group were fed a normal diet, with drinking water containing 0.08 mg/kg methimazole, and those in the DS group were fed a high-fat diet, injected with STZ at the age of 11 weeks, and supplied drinking water containing 0.08 mg/kg methimazole. The body weights and blood glucose levels of the mice were measured at 1-week intervals.

Compared with mice in the NC group, DC mice exhibited a significant increase in body weight (Figure 1(b)). Furthermore, SCH induction significantly increased the body weights of mice in the NS (from 12 weeks, *P* < 0.05) and DS (from 10 weeks, *P* < 0.01) groups compared with those of NC or DC mice. Additionally, compared with the control mice (NC and NS groups), diabetic mice (DC and DS groups) exhibited high FBG levels, which at the age of 12 weeks, reached values above 16.7 mmol/L, indicating that the T2DM model was successfully established (Figure 1(c)). Interestingly, the serum FBG levels of methimazole-induced SCH mice were higher than those in normal-water-drinking mice (*P* < 0.05) in both normal and diabetic mice. Additionally, compared with the controls (NC and DC groups), SCH mice (NS and DS groups) exhibited similar levels of serum FT3 and FT4 levels but showed high TSH levels (Figures 1(f)–1(h)). The glucose tolerance curves corresponding to the DC and DS groups showed considerably attenuated decline in plasma glucose levels after 30 min compared with those in the NC and NS groups, suggesting an obvious impairment of glucose tolerance (Figure 1(d)). The AUCs corresponding to the results of the OGTT showed that SCH induction exacerbated glucose tolerance impairment in both the normal and T2DM groups. Furthermore, HbA1c levels were significantly higher in diabetic mice than in normal mice (Figure 1(e)).

As shown in Figures 1(i)–1(l), the TC, TG, and LDL-C levels of mice in the DC group increased, while their HDL-C levels showed a significant decrease compared with those in NC mice. Additionally, SCH induction resulted in increased concentrations of TC, TG, and LDL-C, while decreasing HDL-C levels, although these were not statistically significant. Figures 1(m)–1(p) indicated elevated insulin and leptin levels in mice in the diabetic group, which also showed significantly higher HOMA-IR indices than NC and NS mice. Conversely, the diabetic group showed lower adiponectin levels, while the DC and DS groups showed no differences in this regard.

### 3.2. Effect of TSH on Diabetic Peripheral Neuropathy

**Figure 2:**
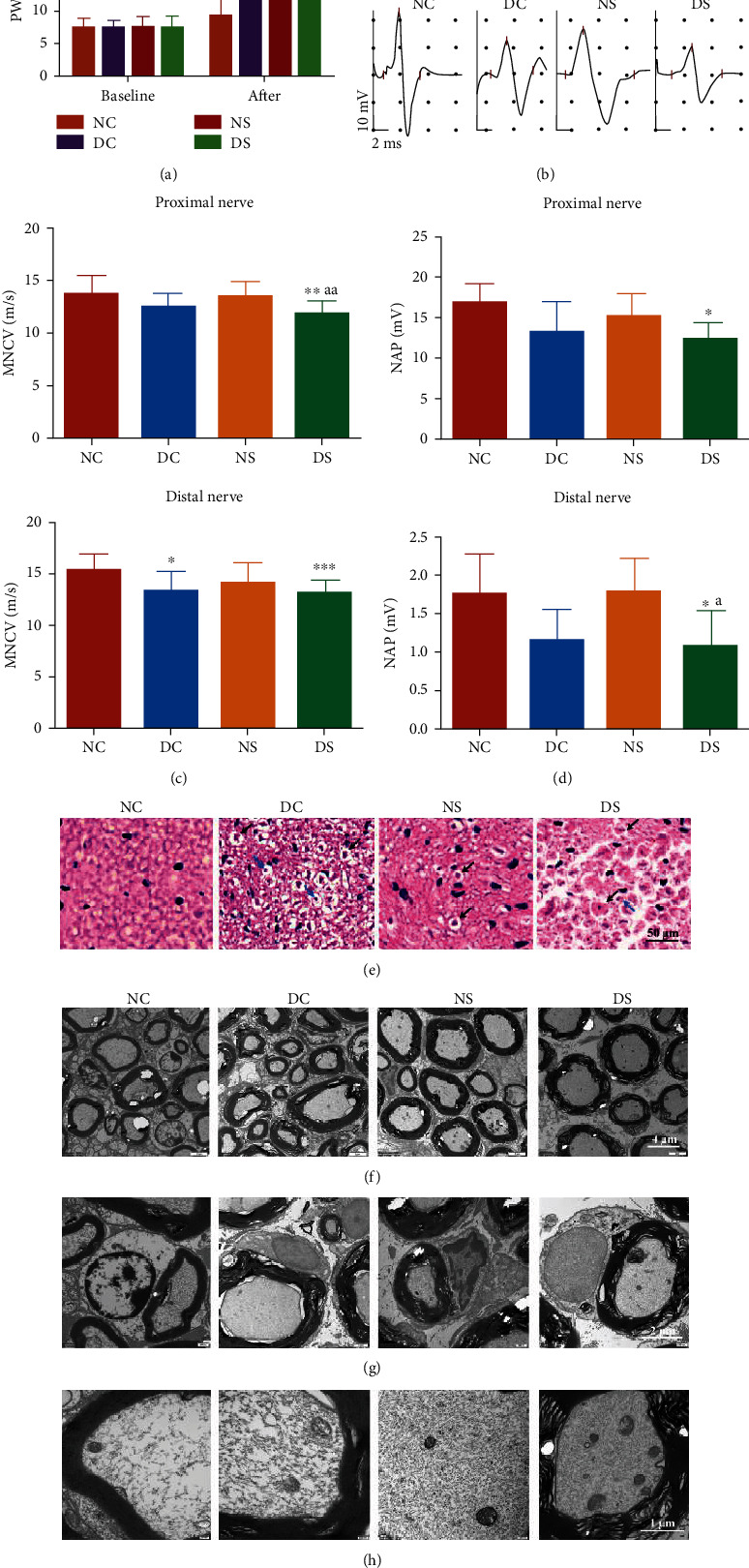
Animal behavior and pathophysiology. (a) Paw withdrawal latency (PWL) owing to heat stimulation. The bar graphs illustrate the results corresponding to the NC, DC, NS, and DS groups at baseline and at 25 weeks after grouping. At baseline, the different mice groups showed no PWL differences in response to heat stimulation (*n* = 7). (b) Representative electroneurogram of mice (*n* = 6). Bar charts showing the comparison of (c) MNCV and (d) NAP corresponding to the proximal and distal nerve, respectively. MNCV: motor nerve conductive velocity; NAP: nerve action potentials. (e) Representative H&E staining images of sciatic nerve cross-section. The sciatic nerves of mice in the DC or DS groups exhibited substantial vacuolar-like defects (black arrows) and axonal shrinkage (blue arrows). Magnification, 400x. (f–h) Ultrastructure of sciatic nerve cross-sections based on TEM. Representative sciatic nerve images of mice in the NC, DC, NS, and DS groups at a magnification of (f) 8000x, (g) 20000x, and (h) 40000x. NC: normal control; DC: diabetic control; NS: normal SCH induction; DS: diabetic SCH induction. All data are presented as mean ± SD, a one-way ANOVA was used to analyze (a, d), and nonparametric Kruskal-Wallis test was used to analyze (c) (^∗^*P* < 0.05, ^∗∗^*P* < 0.01, and ^∗∗∗^*P* < 0.001, compared with the NC group; ^#^*P* < 0.05, ^##^*P* < 0.01, and ^###^*P* < 0.001, compared with the DC group; ^a^*P* < 0.05, ^aa^*P* < 0.01, and ^aaa^*P* < 0.001, compared with the NS group).

PWL, which reflects sensory nerve function in response to heat, did not differ among the mice groups at baseline (Figure 2(a)). However, our results revealed an obvious increase in PWL values in DC mice, indicating impaired sensitivity to pain in diabetes. Further, compared with the NC mice, SCH mice showed an increase in PWL. Meanwhile, the PWL values in DS mice were significantly increased, compared with DC mice, suggesting that TSH resulted in an impairment of sensitivity to pain.

NCV determination is the gold standard for defining peripheral neuropathy in patients with diabetes [17]. Compared with those in the NC group, the MNCV and nerve action potentials (NAP) of mice in the DC group showed a significant decrease; this was indicative of peripheral neuropathy in T2DM (Figures 2(b)–2(d)). Additionally, the MNCV and NAP corresponding to mice in the NS and DS groups were lower than those corresponding to the mice in the NC and DC groups, respectively.

To further determine the destructive effect of TSH on the PNS, alterations in sciatic nerve histology were examined. The cross-section of H&E-stained sciatic nerves showed that nerve fibers were more compact in the NC group than in the DC group (Figure 2(e)). Furthermore, mice in the NS group showed more vacuoles compared with those in the NC group, while DS mice exhibited the most severe sciatic nerve fracture. The DC and DS groups showed obvious nerve fiber myelin fragmentation and axonal shrinkage. These observations were confirmed via TEM (Figures 2(f)–2(h)). The nerve fibers of mice in the NC group were orderly and closely packed, while those of mice in the DC group appeared disrupted and disordered, showing a loose arrangement. Vacuolar flaws and isolated lamellar gaps were also observed in nerve fibers from mice in the DC and DS groups, with the DS group showing substantially more myelin fragmentation and vacuolar-like degradation in the myelin sheath than the DC group (Figure 2(f)). Long-term glucolipotoxicity obviously damaged the myelin sheath and Schwann cells of mice, as previously reported. Additionally, the NS, DC, and DS groups showed compromised Schwann cell integrity, with the DS group showing a more aggravated condition than the NS and DC groups (Figure 2(g)). Under diabetic conditions, the mitochondria of the mice showed significant structural alterations, appearing swollen and fractured or lacking cristae (Figure 2(h)). This condition was most severe in the DS group. Hence, these findings provide strong evidence that the severity of DPN in T2DM is significantly enhanced in SCH mice.

**Figure 3:**
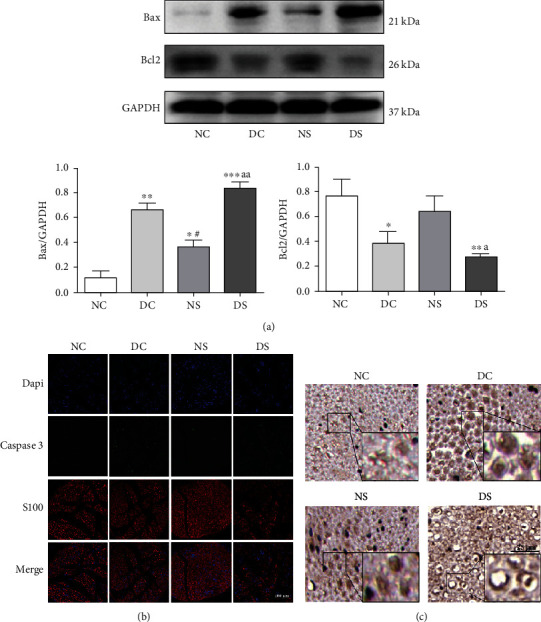
Sciatic nerve apoptosis and TSHR expression in diabetic mice with SCH. (a) Western blotting for determining Bax and Bcl2 expression in the sciatic nerve (*n* = 3). (b) Immunofluorescence results of Caspase 3 expression in the sciatic nerve. (c) IHC of TSHR expression in cross-section of sciatic nerve. TSHR: thyroid stimulating hormone receptor; SCH: subclinical hypothyroidism. All data are presented as mean ± SD (^∗^*P* < 0.05, ^∗∗^*P* < 0.01, and ^∗∗∗^*P* < 0.001, compared with the NC group; ^#^*P* < 0.05, ^##^*P* < 0.01, and ^###^*P* < 0.001, compared with the DC group; ^a^*P* < 0.05, ^aa^*P* < 0.01, and ^aaa^*P* < 0.001, compared with the NS group).

To explore the role of apoptosis in SCH-induced DPN, we examined the expression of several genes implicated in apoptosis regulation, including Bcl2, Bax, and Caspase 3. Bcl2 suppresses apoptosis, while Bax is a proapoptotic member of the Bcl2 protein family. The WB results of sciatic nerve tissue (Figure 3(a)) revealed reduced Bcl2 expression in the DC, NS, and DS groups, and this decrease was the most significant in the DS group. Moreover, Bax expression was lowest in the NC group, moderate in the NS group, and highest in the DS group. These results indicated that sciatic nerve apoptosis was the most serious in T2DM combined with SCH. IHC indicated that the DC and DS groups showed higher TSHR expression than the NC group, with the highest expression level corresponding to the DS group (Figure 3(c)). Additionally, TSHR and Caspase 3 were both expressed in Schwann cells, and the changes in their expression trend were consistent (Figures 3(b) and 3(c)). Given that the existence of a relationship between TSHR and apoptosis in Schwann cells is unclear, further in vitro experiments were performed.

### 3.3. Expression of Functional TSHR by RSC96 Cells

**Figure 4:**
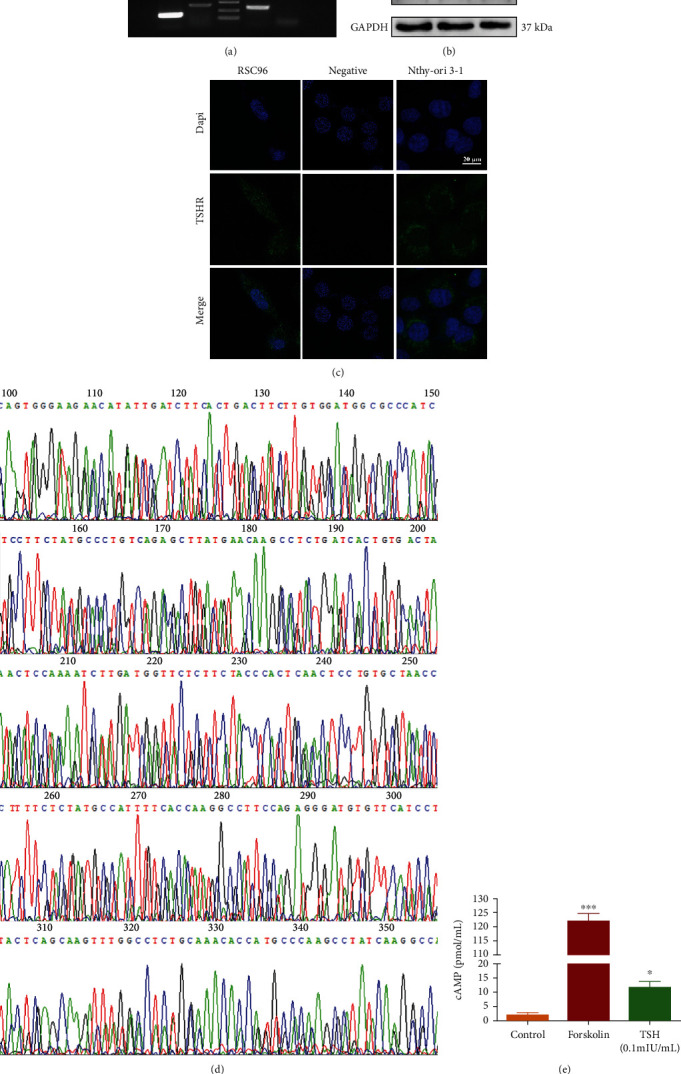
Determination of TSHR expression in RSC96 cells using three distinct methods. (a) TSHR mRNA expression based on PCR. (b) TSHR expression based on WB. Lane 1: HEK293 cells; lane 2: RSC96 cells; lane 3: Nthy-ori 3-1 cells. (c) TSHR expression based on IF assay. The cells were stained with the primary anti-rat TSHR antibody and the FITC-conjugated secondary Ab and Dapi to label the nuclei. (d) Sequencing results of PCR products. (e) Determination of cAMP content in TSH-treated RSC96 cells using an ELISA-based cAMP assay (*n* = 3). Control refers to cells treated only with IBMX. Forskolin treatment served as the positive control for the induction of cAMP production. All data are presented as mean ± SD (^∗^*P* < 0.05, ^∗∗^*P* < 0.01, and ^∗∗∗^*P* < 0.001, compared with the control group).

TSH needs to bind to the receptor TSHR to perform its function. Thus, we conducted experiments using effective methods by which TSHR protein can be detected and visualized. PCR analysis revealed the presence of TSHR mRNA in RSC96 cells (Figure 4(a)). Furthermore, WB revealed that both RSC96 cells and the positive control Nthy-ori 3-1 cells expressed TSHR (Figure 4(b)). The visualization of TSHR via IF revealed its expression in the cell membrane of RSC96 cells (Figure 4(c)). Furthermore, sequencing results showed that the sequence of mice TSHR mRNA was similar to that of human TSHR (Figure 4(d)). Thus, TSHR expression in RSC96 cells was confirmed.

We measured TSH-stimulated cAMP responses to determine the functionality of TSHR observed in RSC96 cells (Figure 4(e)). The results obtained showed that TSH could enhance cAMP production by approximately sixfolds, indicating the functionality of TSHR protein expression in RSC96 cells.

### 3.4. Impairment of Peripheral Nervous Function by TSH, with Decreased Cell Viability and Enhanced Apoptosis in RSC96 Cells

**Figure 5:**
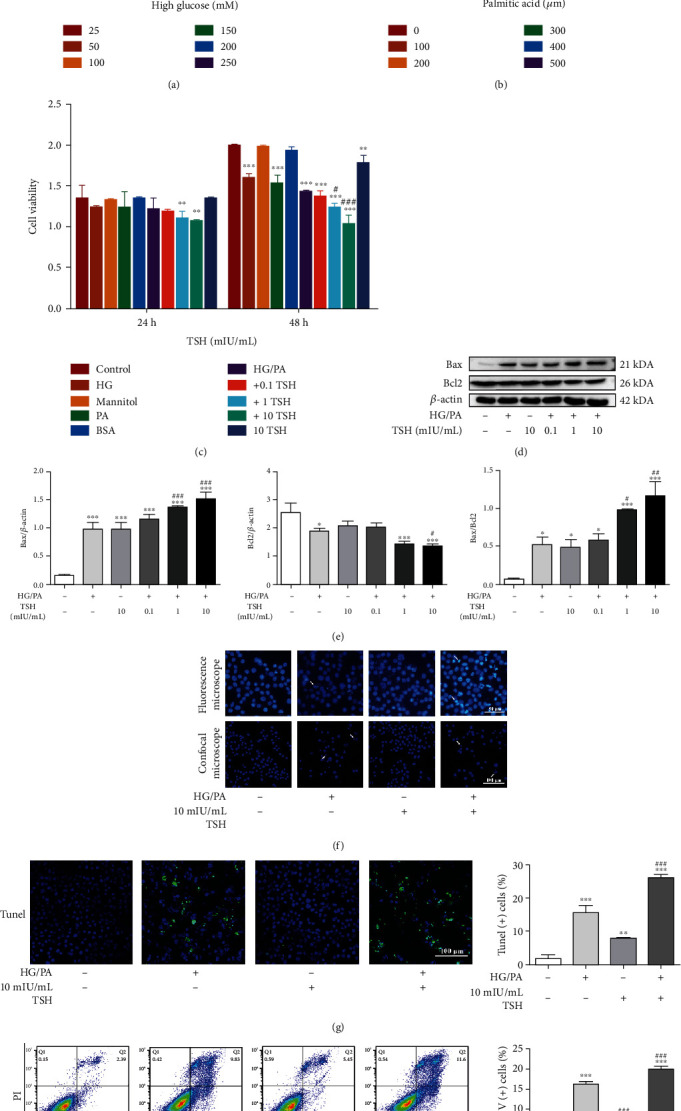
Neurodamage effect of TSH on HG/PA-induced glucolipotoxicity in RSC96 cells. (a) High glucose-induced cytotoxicity (*n* = 3). (b) Cell viability under varied concentrations of palmitic acid (*n* = 3). (c) Cell viability under HG/PA with or without different TSH concentrations. HG: 50 mM high glucose; mannitol: 50 mM mannitol; PA: 300 *μ*M palmitic acid (*n* = 3). (d–e) Bax and Bcl2 protein expression under HG and PA or TSH treatments (*n* = 3). TSH enhanced Bax expression and inhibited Bcl2 expression in a dose-dependent manner. Hoechst staining was performed to determine nuclear morphology. Representative micrographs corresponding to three independent experiments based on fluorescence microscopy or confocal microscopy. (f) Nuclear condensation (white arrows, *n* = 3). Apoptosis detected via (g) TUNEL staining and (h) Annexin V-FITC/PI assay (*n* = 3). All data are presented as mean ± SD (^∗^*P* < 0.05, ^∗∗^*P* < 0.01, and ^∗∗∗^*P* < 0.001, compared with untreated cells; ^#^*P* < 0.05, ^##^*P* < 0.01, and ^###^*P* < 0.001, compared with HG/PA-treated cells).

RSC96 cells were treated with different concentrations of glucose and PA to simulate an in vitro DPN model and evaluate the effect of HG and PA via the CCK-8 assay. Both HG and PA significantly damaged RSC96 cells in a dose- and time-dependent manner, resulting in 17.96% and 46.06% decrease in cell viability following treatment with 50 mM HG and 300 *μ*M PA, respectively (Figures 5(a) and 5(b)). Thus, in subsequent experiments, 50 mM HG and 300 *μ*M PA were used as the optimal damage conditions. Furthermore, we observed that TSH treatment aggravated HG/PA-induced cell viability decline in a dose-dependent manner, and treatment with 10 mIU/mL TSH alone resulted in a significant decreased (10.62%) in cell viability (Figure 5(c)).

Next, we examined the effects of HG/PA treatment with or without TSH on apoptotic gene protein levels and found that 50 mM HG/300 *μ*M PA treatment upregulated Bax protein expression levels, which further increased in HG/PA+TSH-treated cells in a dose-dependent manner (Figures 5(d) and 5(e)). Correspondingly, Bcl2 protein levels were downregulated in HG/PA-treated cells and further decreased in HG/PA+TSH-treated cells. Hoechst staining was performed to visually clarify Schwann cell apoptosis in vitro. As illustrated in Figure 5(f), numerous cell nuclei in the HG/PA-treated group and the HG/PA+10 mIU/mL TSH-treated group exhibited condensed fragments and apoptotic bodies, whereas the untreated cells showed normally dispersed chromatin and intact nuclear membranes. Additionally, TUNEL analysis revealed that the degree of apoptosis in HG/PA+TSH-treated RSC96 cells was much more pronounced than that in HG/PA-treated cells (Figure 5(g)). The Annexin V-FITC/PI assay (Figure 5(h)) showed that the proportion of apoptotic cells in the HG/PA-treated mice group increased considerably from 4.273% to 16.3% and further increased to 19.97% following the addition of TSH. These findings indicated that the elevated TSH levels significantly impaired RSC96 cell function and increased apoptosis in vitro.

### 3.5. Aggravation of Oxidative Stress and Mitochondrial Dysfunction in RSC96 Cells by TSH under High Glucose and Palmitic Acid Conditions

**Figure 6:**
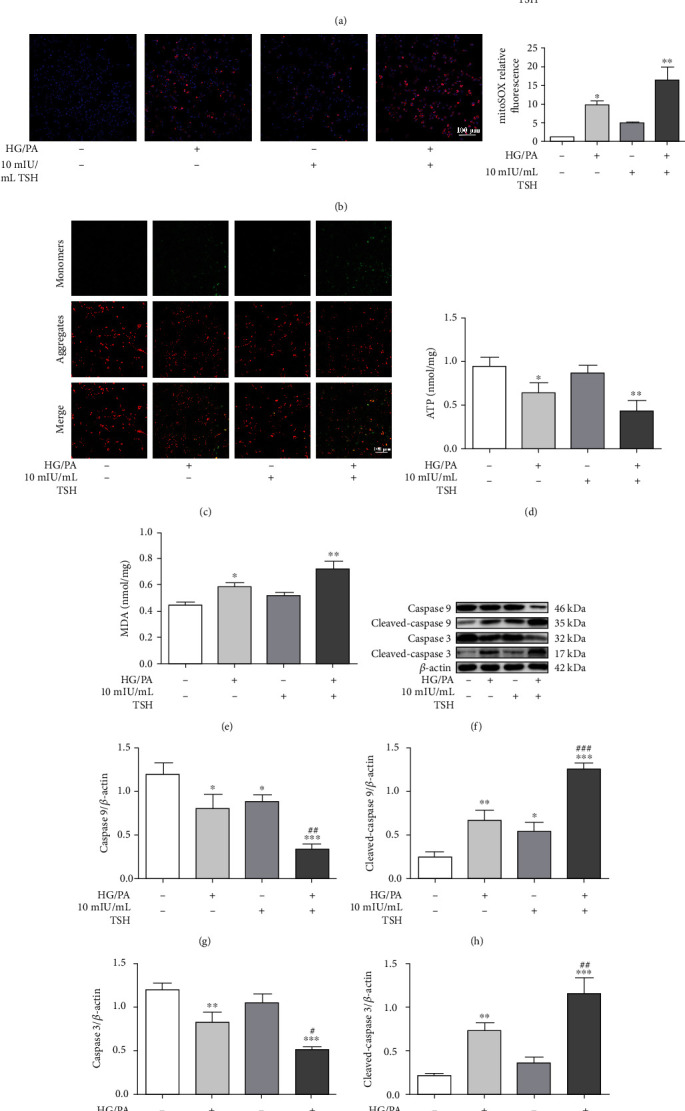
Aggravation of oxidative stress and mitochondrial dysfunction in HG/PA-injured RSC96 cells following TSH treatment. (a) Effect of TSH on ROS levels (*n* = 3). (b) Mitochondrial ROS production in response to different treatments detected via MitoSOX staining (*n* = 3). (c) Mitochondrial membrane potential (MMP) analysis. MMP levels were visualized via fluorescence microscopy (*n* = 3). Effects of TSH on (d) ATP and (e) MDA levels in RSC96 cells (*n* = 3). Representative Western blotting bands (f) and quantitative analysis (g–j) of Cleaved-caspase 9 and Cleaved-caspase 3 in RSC96 cells (*n* = 3). All data are presented as mean ± SD (^∗^*P* < 0.05, ^∗∗^*P* < 0.01, and ^∗∗∗^*P* < 0.001, compared with untreated cells, ^#^*P* < 0.05, ^##^*P* < 0.01, and ^###^*P* < 0.001, compared with HG/PA-treated cells).

To investigate whether cell apoptosis caused by TSH on RSC96 cells occurred through an ROS-mediated mechanism, ROS generation and lipid peroxidation were assessed. Figures 6(a) and 6(e) show that HG/PA stimulation elevated intracellular ROS production and MDA levels compared with that in untreated cells. Furthermore, treatment with 10 mIU/mL TSH significantly increased ROS production and MDA levels compared with HG/PA treatment.

We investigated the effect of TSH treatment on mitochondrial ROS production, MMP, which reflects mitochondrial function, ATP levels, and protein expression so as to clarify whether TSH targeted mitochondrial function. Both HG/PA- and TSH-only treated cells showed more mitochondrial ROS generation than untreated cells, with high MitoSOX fluorescent presentation (Figure 6(b)). Further, TSH markedly enhanced MitoSOX staining in HG/PA-treated RSC96 cells. As shown in Figure 6(c), both HG/PA- and TSH-only treatments as well as HG/PA+TSH treatment showed enhanced green fluorescence and decreased red fluorescence compared with untreated cells. The reduced red/green fluorescence ratio indicates a loss of MMP and mitochondrial depolarization. However, the HG/PA+TSH-treated cells and the TSH-only treated cells showing the most and least severe reduced red/green fluorescence ratio, respectively. Decreased ATP levels are also indicative of impaired or reduced mitochondrial function. Our results indicated that ATP levels decreased after HG/PA injury, with TSH treatment further inducing a decline in ATP levels in the RSC96 cells (Figure 6(d)).

In this study, the expression levels of mitochondrial apoptosis-related proteins, such as Caspase 9 and Caspase 3, were examined (Figures 6(f)–6(j)). HG/PA-treated cells as well as TSH-only treated cells showed increased Cleaved-caspase 9 and Cleaved-caspase 3 expression levels. Moreover, TSH treatment exacerbated HG/PA-induced apoptosis given that Cleaved-caspase 9 and Cleaved-caspase 3 expression levels were enhanced in HG/PA+TSH-treated cells. These results suggest that TSH further aggravated oxidative stress and mitochondrial dysfunction in RSC96 cells induced by high glucose and PA.

### 3.6. TSHR Knockout Protects RSC96 Cells from the Damaging Effects of TSH

**Figure 7:**
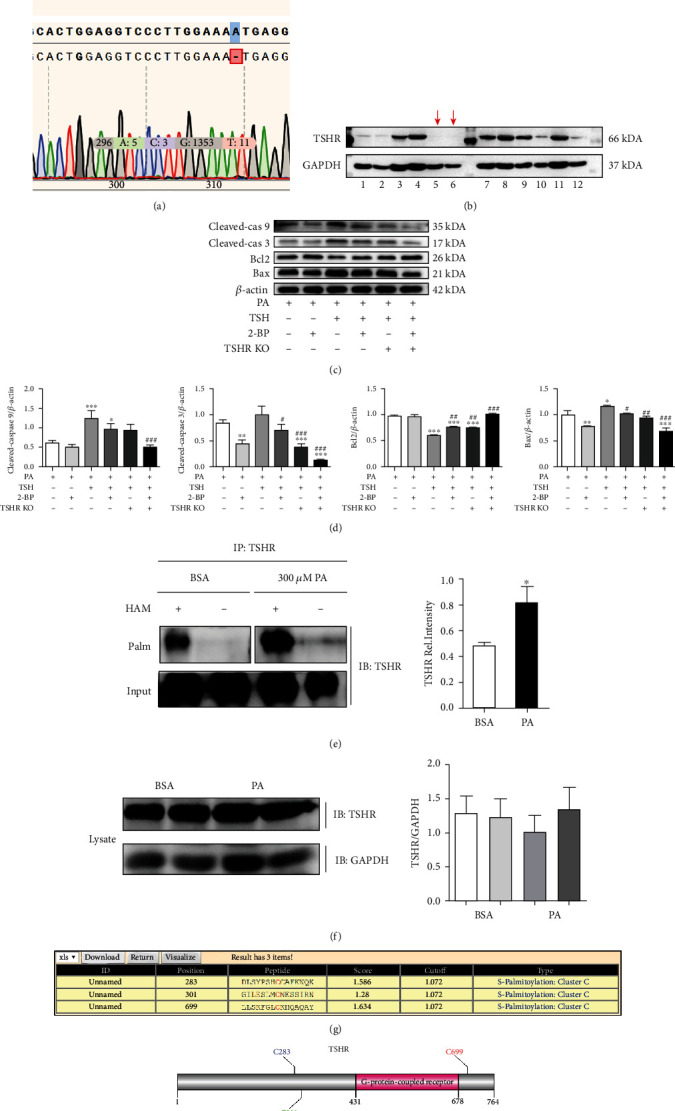
Promotion of TSHR palmitoylation by palmitic acid, leading to the enhancement of the proapoptotic effects of TSH. (a) Sequencing results of the gene missing a base. Deletion of base A caused frameshift mutations and premature termination of translation that could not be translated into the TSHR protein. (b) WB results corresponding to cultured monoclonal cell lines. Each band represents a monoclonal cell line. The red arrows are indicative of stable TSHR knockout cell lines. (c) Expression levels of Cleaved-caspase 9, Cleaved-caspase 3, Bcl2, and Bax determined via western blotting (*n* = 3) and (d) analyzed. Cleaved-cas 9: Cleaved-caspase 9; Cleaved-cas 3: Cleaved-caspase 3 (^∗^*P* < 0.05, ^∗∗^*P* < 0.01, and ^∗∗∗^*P* < 0.001, compared with the PA-treated cells; ^#^*P* < 0.05, ^##^*P* < 0.01, and ^###^*P* < 0.001, compared with PA+TSH-treated cells). (e) S-palmitoylation levels of TSHR after PA treatment. HAM: hydroxylamine; Palm: palmitoylation; IP: immunoprecipitation; IB: immunoblotting. ^∗^*P* < 0.05, ^∗∗^*P* < 0.01, and ^∗∗∗^*P* < 0.001, compared with the BSA-treated cells. (f) TSHR expression in cell lysates. There was no significant difference of TSHR expression in cell lysates. (g) Predicted palmitoylation sites in TSHR using GPS-lipid. (h) Visualization of the predicted lipid modification sites. All data are presented as mean ± SD.

To clarify the molecular mechanism underlying TSH-induced apoptosis, TSHR knockout RSC96 cells were established.  Figure 7(a) showed the target gRNA sequence and sequencing results corresponding to the successful removal of the base, resulting in the generation of an early termination codon due to frame shift mutation.  Figure 7(b) showed the results of WB corresponding to the culturing of TSHR knockout monoclonal cell lines. The stable and successfully established TSHR knockout cell lines are indicated using red arrows. Specifically, TSHR knockout significantly decreased TSHR expression. This resulting TSHR deficiency attenuated TSH-triggered apoptosis in RSC96 cells, which, correspondingly, exhibited decreased Bax, Cleaved-caspase 3, and Cleaved-caspase 9 expression, and increased Bcl2 expression (Figures 7(c) and 7(d)).

### 3.7. Promotion of TSHR Palmitoylation by Palmitic Acid Leading to the Enhancement of the Proapoptotic Effects of TSH

Reportedly, TSHR has intrinsic palmitoylation properties. Thus, we hypothesized that PA can enhance TSHR palmitoylation and hence augment the proapoptotic effect of TSH. Accordingly, we investigated the S-palmitoylation level of TSHR via the hydroxylamine restoration assay. Results indicated that the protein bands of PA-treated cells were significantly stronger than those of untreated cells, indicating that PA could significantly promote the palmitoylation level of TSHR (Figure 7(e)). However, the lysates showed no significant differences with respect to TSHR expression (Figure 7(f)).

The palmitate analog, 2-BP, is the most commonly used palmitoylation inhibitor in cells. Pretreatment with 2-BP for 6 h reduced the expression levels of Bax, Cleaved-caspase 3, and Cleaved-caspase 9, while enhancing those of Bcl2 compared with PA treatment (Figure 7(c)). These findings suggest that palmitoylation plays a role in the promotion of TSH-induced apoptosis. Further, three sites with high palmitoylation scores in TSHR were determined, with site 699 being the most probable palmitoylation site (Figure 7(g)), as shown in Figure 7(h).

## 4. Discussion

Whether TSH exacerbates DPN in patients with DM remains uncertain. In this study, we report for the first time that T2DM mice with SCH (DS group) show more severe peripheral neuropathy than normal mice (NC group) or diabetic mice without SCH (DC group), with slower NCV and more severe myelin fragmentation, vacuolar flaws, and isolated lamellar gaps. Further, compared with NC mice, the other three mice groups showed increased TSHR and Caspase 3 expression, with the DS group showing the highest TSHR expression level. Based on in vitro experiments, we further established that Schwann cells express functional TSHR. We also observed that elevated TSH levels enhanced HG/PA-induced oxidative stress and apoptosis of RSC96 cells. Thus, our findings also provide insight into the critical role of palmitoylation in PA-induced perturbations of PNS. Given that TSH acts by binding to TSHR, we established TSHR knockout RSC96 cells and used a crisper-cas9 assay to elucidate the molecular mechanism underlying TSH-induced apoptosis. Our results indicated that TSHR knockout significantly inhibited apoptosis, and TSHR palmitoylation, which was enhanced by PA treatment, possibly played an important role.

In our study, mice in the NS group showed abnormal FBG levels and HbA1c expression, an observation that is consistent with that reported in previous studies indicating the existence of a significant positive correlation between TSH levels and FBG or HbA1c levels in patients with T2DM [18–20], whereas other studies have reported that TSH has no effect on FBG levels in healthy people [21]. Additionally, the majority of studies lean towards the stance that high normal serum TSH levels are associated with T2DM incidence [22, 23] and may be an additional contributor to the incidence of this disease [24–26]. In this study, the DC mice group showed increased TSH levels, an observation that is consistent with that reported by Liu [27]. Our results suggested that T2DM and SCH, which are common endocrine disorders, interact and frequently coexist. Furthermore, T2DM shares an underlying pathology with SCH, and several possible mechanisms that explain this link have been reported. A potential mechanism might be the complex interaction between the signaling pathways associated with glycometabolism and insulin resistance [28–30] and hyperlipidemia [31] in T2DM, with higher serum TSH levels. Consistent with this possible mechanism, our investigations showed elevated lipid levels in NS mice, even though no significant differences in insulin resistance were observed. These results suggest a complex and intersecting clinical outcome for the relationship between TSH and insulin resistance or T2DM.

Animal behavior results suggested that peripheral neuropathy in the DC group was more severe than that in the NC group. This has recently been confirmed in several basic studies [32, 33]. We further confirmed that diabetic mice with SCH (DS group) showed more severe peripheral neuropathy than diabetic mice without SCH, indicating that SCH conditions have damaging effects on PNS and may increase the risk of DPN, which, possibly, can be attributed to the direct effects of TSH on PNS, as thyroid hormone levels in SCH mice were normal. According to the results of previous studies, TSH levels are substantially associated with DPN and are an independent risk factor and a useful predictor for DPN [11–13]. This is consistent with our research results. The results of TEM and IHC in this study also supported this theory, suggesting that TSH plays an important role in aggravating DPN.

DPN is usually characterized by loss of myelin sheath integrity and Schwann cell apoptosis, and it is well known that long-term exposure to hyperglycemia conditions and lipids is a key contributor to the onset of DPN [34, 35]. Here, we established an HG/PA-injured cellular model using rat RSC96 cells to mimic the in vitro DPN model and discussed the relationship between RSC96 cells and glucose, lipid, and TSH levels, as well as the mechanism of TSH-induced Schwann cell apoptosis to the end of providing theoretical evidence for DPN etiology [36]. Maintaining the function and integrity of Schwann cells is important in DPN treatment. Our TEM results revealed that the Schwann cells of mice in the DC and NS groups were disrupted and appeared structurally incomplete, with the DS group showing the most severe degree of disruption. Another study confirmed the destruction of Schwann cells in DPN mice based on observations made via electron microscopy [32]. In this study, our in vitro experiments demonstrated that TSH further impaired the viability of RSC96 cells injured in response to HG/PA treatment. These findings are consistent with the behavioral neurological damage in T2DM mice with SCH, indicating that the damaging effect of TSH on Schwann cells possibly promotes DPN.

The function of TSH is mediated through TSHR, which is expressed in a variety of extrathyroidal cells, such as adipocytes [37, 38], osteoclasts [39], hepatocytes [40, 41], and macrophages [26]. For the first time, we investigated whether Schwann cells expressed TSHR. Our results showed that TSHR was functionally expressed in RSC96 cells, and this TSHR expression was primarily concentrated in the Schwann cell membrane region based on IF assay. This observation supports our hypothesis that TSH can affect Schwann cells in the PNS.

The IF experiments performed in this study revealed increased Caspase 3 expression in the sciatic nerve, and in vitro experiments showed that TSH could further enhance the apoptosis of RSC96 cells owing to treatment with high glucose and high PA concentrations. Previous studies have shown that TSH promotes apoptosis in different cells. Specifically, based on cDNA array hybridization, Brokken et al. revealed that most of the genes regulated by TSH are related to cell apoptosis [42]. Further, Xin et al. reported that TSH stimulation upregulates Bax expression and downregulates Bcl2 expression, thus disrupts the balance between Bax and Bcl2, while promoting chondrocyte apoptosis [43]. It has also been reported that TSHR upregulation promotes the apoptosis of thyroid cancer cells [44].

The results of this study indicated that elevated TSH levels enhance ROS production in RSC96 cells and may trigger oxidative stress. Previous studies have reported elevated oxidative stress in SCH individuals [13], which is considered to be positively associated with TSH levels [45]. These previous reports are consistent with our results. Further, MDA is a lipid peroxidation marker that is used to assess lipid peroxidation owing to increased oxidative stress. In this study, TSH resulted in an increase in MDA levels, confirming increased oxidative stress. Mitochondria are important sites and key regulators of redox homeostasis and cellular functions, and an imbalance in the mitochondrial redox status results in excessive mitochondrial ROS production [45]. Haribabu et al. found that excessive TSH levels directly induce oxidative stress. Additionally, Liu et al. reported that increased TSH levels inhibit the AMPK/SIRT3 signaling pathway in endothelial cells, resulting in increased mitochondrial permeability transition pore opening, reduced electron transport chain activity, and increased mitochondrial ROS production [36]. Consistent with the results of this study, our findings also established a connection between TSH and Schwann cell mitochondrial dysfunction based on the observed increased in ROS generation. MitoSOX fluorescence enhancement further confirmed that TSH enhances mitochondrial ROS production. Additionally, mitochondrial dysfunction in RSC96 cells injured owing to TSH treatment was characterized by decreased MMP and ATP production. Indeed, our results suggested that TSH exacerbated HG/PA disturbed-mitochondrial dysfunction in RSC96 cells and that mitochondrial ultrastructure was significantly impaired in the sciatic nerve of DC and DS mice, with the condition being worse in DS mice.

TSHR knockout in RSC96 cells via the crisper-cas9 method resulted in decreased apoptosis, indicating that treatment with elevated TSH levels enhanced apoptosis in RSC96 cells. Notably, in this study, the DC, NS, and DS mice groups showed increased TSHR expression compared with NC mice. The potential mechanism for this observation may involve low thyroid function. Further, the relatively low levels of thyroid hormones in DC mice possibly resulted in decreased thyroglobulin levels, hence the decrease in the inhibition of TSHR transcription [46]. Second, autoimmunity has been identified as the primary etiology of thyroid-dysfunction-associated DM [47, 48]. Additionally, the action of various cytokines in diabetes induces thyroid autoimmunity, and the combination of TRAb produced with the TSHR-*α* subunit prevents its conformation from changing, thus delaying its shedding, and the half-life of TSHR is prolonged [49].

Finally, we observed that DPN in mice with T2DM and SCH was more severe than that in mice with T2DM or SCH alone. Additionally, the combined effect of TSH and PA treatments was significantly stronger than their individual effects. Therefore, the mechanism by which TSH potentiates HG/PA injury may be related to the PA-induced increased in TSHR palmitoylation, which affects the conformation of several G protein-coupled receptors (GPCRs) and regulates downstream signal transduction, intracellular transport, and oligomerization owing to interactions between GPCRs, G proteins, and lipid molecules. Reportedly, PA can modify the palmitoylation of several proteins. Specifically, the PSD-95 protein is palmitoylated by PA, which controls its affiliation with cell membranes [50]. Further, Wnt protein requires palmitoylation by PA for proper intracellular transport during secretory processes [51]. Tanaka et al. also reported that palmitoylation may enhance the rate of intracellular TSHR trafficking, while its abolition may delay cell surface TSHR expression [52]. In this previous study, cysteine-699 was identified as the lipid modification site of TSHR. This observation is consistent with our predicted value, with the 699-position showing the highest score. Our results also strongly suggested that palmitoylation enhances the affinity of TSHR protein tethering to Schwann cell membranes. This possibly enhances TSHR expression in the cell membrane and further activates the downstream apoptotic pathway. Similar to the results of our study, Galluzzo et al. observed that ER*α* palmitoylation promotes the localization of ER*β* in the cell membrane, initiating downstream apoptotic signaling and inhibiting the proliferation of colon cancer cells [53].

Our study demonstrated for the first time through mouse and in vitro experiments that Schwann cells express TSHR and that TSH may enhance oxidative stress and apoptosis in Schwann cells by binding to TSHR, thereby revealing the mechanism by which diabetes combined with SCH exacerbates DPN. These findings have implications for clinical guidance with respect to enhancing the management of DM combined with SCH. However, one limitation in this regard is that we used cell lines instead of primary Schwann cells and did not explore the effect of TSH on neurons, given that damage to neurons may affect DPN to some extent. Another limitation is that the specific downstream signaling pathways are still unclear. Therefore, further investigations are needed to fully elucidate the detailed underlying mechanisms.

## 5. Conclusions

In conclusion, our in vivo and in vitro findings revealed that TSH can exacerbate abnormal glucose and lipid metabolism and significantly aggravate DPN. The underlying mechanisms possibly include Schwann cell oxidative stress and apoptosis owing to TSHR palmitoylation. Furthermore, the results of this study demonstrate that TSHR is a potential target for both the prevention and treatment of DPN and, possibly, other microvascular diseases.

## Figures and Tables

**Table 1:** Primers used for TSHR DNA amplification.

Gene	Primer	Sequence (5′-3′)	Expected product (bp)
Rat TSHR 1	Forward	AAGCTGGATGCTGTTTACCT	167
	Reverse	GTTCTTCGCGATCAGCTCTT	
Rat TSHR 2	Forward	ACATCGCCCTTGTTCTCCTG	385
	Reverse	TCTCTGGGCCTGATAGGCTT	
Human TSHR 1	Forward	CCTCTCATCACTGTTAGCAA	324
	Reverse	TACTCTTCTGAGATTTGGCC	
Human TSHR 2	Forward	TTTGACAGCCATTATGACTACACC	839
	Reverse	TTGGAGTTGCTAACAGTGATGAGA	

## Data Availability

The data used to support the findings of this study are available from the corresponding author upon request.
